# Kinetics of molecular patterns captured by mannose-binding lectin in septic shock correlate with clinical outcome: a monocentric prospective observational study

**DOI:** 10.1186/s40635-025-00832-x

**Published:** 2025-12-10

**Authors:** Alain Bicart-See, Fanny Vardon, Stephanie Ruiz, Jean-Marie Conil, Veronique Tudal, Michael Super, Donald E. Ingber, Eric Oswald

**Affiliations:** 1 Institut de Recherche en Santé Digestive (IRSD), INSERM U1220-INRA U1416-ENVT, Université Toulouse III - Paul Sabatier, Toulouse, France; 2 Hôpital Joseph-Ducuing, 15 Rue Varsovie, 31300 Toulouse, France; 3 Critical Care Unit, Anesthesiology and Critical Care Department, Toulouse University Hospital, 1 Av du Pr J Poulhes, 31400 Toulouse, France; 4 Wyss Institute for Biologically Inspired Engineering at Harvard University, 201 Brookline Ave., Boston, MA 02215 USA; 5 Service de Bactériologie-Hygiène, Centre Hospitalier Universitaire de Toulouse, Hôpital Purpan, Toulouse, France

**Keywords:** Mannose-binding lectin (MBL), FcMBL, Septic shock, Pathogen-associated molecular patterns, Biomarker, Innate immunity, Sequential Organ Failure Assessment (SOFA) Score

## Abstract

**Background:**

Various serum biomarkers and scoring systems are currently employed to manage septic critically ill patients. However, a paucity of biomarker evidence facilitates sepsis identification or prognosis. Mannose-binding lectin (MBL) is the main circulating protein in innate immunity. It acts as a broad-spectrum recognition molecule that binds most pathogens, along with their breakdown products and cell debris. We report results of an original approach dosing molecular patterns captured by FcMBL, an engineered version of MBL, in patients with septic shock. This study aimed at evaluating molecular patterns kinetics to assess their potential contribution to the clinical management of critically ill patients suffering from septic shock.

**Results:**

This monocentric, prospective, observational study was conducted on adults admitted to the intensive care unit (ICU) for septic shock. Using magnetic microbeads coated with FcMBL, we quantified molecular patterns captured in blood and analyzed their kinetics for 5 days. Pathogen-associated molecular patterns (PAMP) levels were sampled at 6-h intervals over the first 24 h of ICU admission, then at 12 h intervals on Day 2, and then daily through Day 5. To align the data from the real time of admission to the ICU, the “Serial Measurements” module in MedCalc^®^ software enabled the incorporation of advanced methods, such as mixed models. Outcomes were the persistence of sepsis after Day 5 and adherence to routine sepsis metrics. Thirty-nine patients were included in the study. At Day 5, 21 patients had recovered from sepsis with a sequential organ failure assessment (SOFA) score < 2, while 18 were not. The initial values of PAMP yielded a median concentration of 5 ng/mL. The peak concentration was observed at 9 ng/mL, with a median delay of 24 h. Significant differences were observed in kinetic curves according to the SOFA score at Day 5, with a notable delay in time to peak (Tmax) for the prolonged sepsis group (Hour 48) compared to the short-term sepsis group (Hour 18) (p < 0.001). Compared to standard biomarkers, Tmax PAMP was the most discriminative factor for an unfavorable outcome.

**Conclusions:**

Molecular pattern levels captured by FcMBL during septic shock exhibited large inter-patient variability, suggesting values depend on numerous parameters. The signal’s kinetics demonstrated predictive value and may contribute to clinical management.

*Trial registration*: clinicaltrials, NCT03457038, Registered 15 October 2017, https://clinicaltrials.gov/study/NCT03457038

## Background

Sepsis remains a leading cause of mortality and long-term morbidity worldwide, often driven by an imbalanced and heterogeneous immune response to infection [1]. While current biomarkers such as C-reactive protein (CRP), procalcitonin (PCT), and lactate help assess severity, they frequently fail to reflect the underlying complexity of host–pathogen interactions in real time. There is a pressing need for novel, dynamic biomarkers that more accurately capture disease trajectory and prognostic outlook [2, 3].

Mannose-binding lectin (MBL) is a soluble pattern recognition molecule central to the innate immune system. It binds to a wide array of pathogen-associated molecular patterns (PAMPs), as well as cellular debris and glycosylated ligands released during tissue injury [4]. The release of PAMPs can occur spontaneously and increases significantly following cell lysis by antimicrobial agents or immune cell response [5]. MBL also binds endogenous molecules released during inflammatory stress, named damage-associated molecular patterns (DAMPs) [6, 7].

An engineered version of MBL (FcMBL) has been developed for diagnostic and therapeutic applications, including blood purification and pathogen detection in sepsis [8]. The FcMBL is a chimeric protein formed from the MBL neck and the carbohydrate recognition domain fused to the Fc portion of human IgG1. In parallel, an MBL-based enzyme-linked immunosorbent assay (ELISA) was developed to detect and quantify levels of FcMBL-captured PAMPs in whole blood from septic patients. This ELISA distinguished infection from trauma-related inflammation in a cohort of patients [9].

PAMP kinetics during septic shock has not been well studied to date [10]. Using this ELISA, we investigated how FcMBL’s broad affinity for microbial and endogenous molecular patterns could serve as a biomarker of prognosis for patients with septic shock.

## Methods

### Study design

We conducted a monocentric, prospective, non-randomized, observational study of PAMP kinetics in adults admitted for septic shock. The study was conducted in the intensive care unit (ICU) of Rangueil Hospital, Centre Hospitalier Universitaire de Toulouse, France.

Inclusion criteria were adult patients hospitalized in the ICU for septic shock. Non-inclusion criteria were patients receiving long-term immunosuppressive therapies, including immunosuppressive agents after solid organ transplantation, criteria related to regulation, patients who decline participating in the assay.

Septic shock criteria were defined according to Sepsis-3 [3]. Patients were monitored using standard management for patients with septic shock. PAMPs were sampled as soon as possible after admission in ICU, then every six hours over 24 h, then every 12 h the second day, and then daily until Day 5.

Formal ethical approval was obtained by the institutional review board (IRB), Nº RC/31/17/0157 and declared to the National Commission for Data Protection and Liberties. Informed consent was obtained for all included study patients or relatives if necessary.

We also measured PAMP levels from waste blood samples in emergency department (ED) patients admitted for noninfectious conditions. Waste samples from patients associated with previously published data allowed us to determine the threshold with septic shock patients [9]. Their blood PAMP values were compared with those obtained at the first draw from patients in septic shock.

### Outcomes

Primary outcome of our study was to assess the prognostic value of PAMPs in patients with septic shock. For this pilot clinical study, we assessed that short-term mortality was not a sufficiently discriminating criterion. Indeed, mortality at Day 30 is one of the strongest prognostic markers in intensive care. However, studying this criterion requires a large sample size, especially as mortality rates are becoming increasingly lower thanks to the implementation of the Surviving Sepsis Campaign and the improvement in the overall management of our patients.

Given the size of our cohort and the expected low mortality rate, we evaluated the impact of this biomarker on the patient's outcome using the SOFA score. Organ dysfunction score was assessed using the daily SOFA score. Short and prolonged sepsis were differentiated according to the SOFA score at Day 5 or to the last score for patients who were discharged earlier. Short sepsis were patients with a last SOFA score < 2 within 5 days. Prolonged sepsis were patients maintaining a SOFA score ≥ 2 at Day 5.

Secondary outcomes were to investigate the relationship between PAMP levels and microbiological data, and main serum biomarkers during the first five days of septic shock. PAMPs kinetics were compared with blood culture, lactate, C-reactive protein (CRP), and procalcitonin (PCT).

### Extraction and detection of PAMPs

FcMBL is biotinylated for oriented attachment to streptavidin-coated magnetic beads. There are approximately 36,000 FcMBL molecules per bead, and a final MBL concentration on beads of 5 mg/mL.

Blood samples for PAMPs dosage were collected in heparin tubes and treated within 24 h.

The FcMBL-based ELISA leverages the engineered FcMBL previously described [9].

Each assay contained a mannan standard curve from 0 to 32 ng/mL. PAMPs’ concentration was expressed relative to these internal mannan dilution curves. All measures were triplicated.

### Statistical analysis

Distribution of the values was checked with the Kolmogorov–Smirnov, and kurtosis and skewness assessments. Results were expressed among type of distribution using mean, standard deviation, or median with interquartile range. Qualitative variables were expressed as number and percentage.

The two groups were compared using non-parametric tests, including Mann–Whitney for continuous variables and chi-squared or Fisher’s exact tests for nominal variables. Repeated measures analysis of variance, Friedman and post hoc tests were used to compare within-subject effects. We used the Kruskal–Wallis test to compare our two groups to control patients. In analyses involving multiple comparisons, to minimize the risk of Type I error, we applied appropriate post hoc tests (Dunn’s test or Conover’s test, as applicable).

Clinical constraints occasionally affected the timing of the first PAMP sample. To address this, the statistical analysis was adapted to account for the variability in sampling times. Specifically, the dataset was reconstructed to achieve a uniform time reference from ICU admission and vasopressor initiation. Analyses were performed using a mixed model logistic regression. The “Serial measurements” module in MedCalc^®^ software was applied, with data entered individually for each patient. The software automatically selected the appropriate statistical tests for repeated measures according to the distribution of the parameters. This approach ensures that the analysis is reproducible in accordance with the MedCalc^®^ software terms of use for the “Serial measurements” module. Serially measured parameters were analyzed for each patient using minimum (Cmin) and maximum concentrations (Cmax), time to peak (Tmax), last–first value difference, percentage of time related to threshold, and time-weighted average.

Discriminative value of the variables of interest with respect to clinical judgment parameter was evaluated with receiver-operating-characteristics (ROC) curves, prioritizing those with an area under the curve (AUC) ≥ 0.8 or including this value within a 95% confidence interval. The choices for the most discriminatory thresholds were based on the best Youden index.

Relationships between variables were analyzed using linear regression or Spearman’s rank correlation. The study was conducted using MedCalc® statistical software version 20, with a significance level of p < 0.05 considered statistically significant.

## Results

A total of 39 patients with septic shock were included in the study from October 2018 to May 2023, though recruitment was interrupted from April 2020 to January 2022 due to the Covid pandemic. An IRB extension was accepted in January 2022 and 8 more patients were included.

Recruitment disruption did not allow us to reach the initial target cohort of 50 patients.

Overall, despite the temporal interruption, PAMP kinetics in post-COVID patients were consistent with the patterns observed in the pre-COVID cohort. General characteristics of patients according to Covid interruption are presented in Additional Table 2. The only significant difference identified between these 8 patients compared to the 31 included before the Covid pandemic, was a longer ICU length of stay, 17 days versus 10 (p = 0.011).

Two of them had a Covid infection at inclusion, one with a secondary *Streptococcus pneumoniae* and *Staphylococcus aureus* lung infection, and one with a duodenal perforation. Investigators, techniques used, reagents, controls were the same before and after interruption.

Reasons for non-inclusion included indeterminate shock at enrollment, prior organ transplantation, regulatory criteria (e.g., unaffiliated with the French social security system, inability to sign the informed consent for a relative), or overwhelming clinical workload that limited availability to dedicate to study enrollment. Except for the predefined exclusion criteria, reasons for non-inclusion were not systematically quantified in this study.

### General characteristics

Patients had a median age of 67 years (30–92) and included 30 men and nine women. The origin of infection at inclusion was mainly gastrointestinal (21/39). The 21 septic shocks of digestive origin were 12 times due to gastrointestinal perforation or digestive translocation, including 3 supra-mesocolic. The other digestive origins were 7 acute cholangitis, one infected pancreatitis, one infected cirrhosis and one following hepatectomy for cholangiocarcinoma. Blood cultures sampled at admission to the hospital were positive for 21 patients (54%). In blood cultures, the bacteria isolated were *enterobacteriaceae* in 14 patients, *streptococci* in 7, anaerobic in two, *staphylococcus aureus* in one. Polybacterial blood cultures were identified in 5 patients.

In blood cultures, no fungemia was identified.

In infected tissue from gastrointestinal infections, fungi were isolated in 7 patients, predominantly *Candida albicans*. Regarding bacterial pathogens at the sites of infection, we observed *enterobacteriaceae* in 16 patients, *streptococci* in 9, anaerobic bacteria in 5, non-fermenting bacteria in 3, *staphylococci* in 2. Viruses were identified in 3 patients.

At Day 5, 21 patients had a SOFA score < 2 and a decrease of two points or more. Nine of them were discharged from the ICU. And 18 patients remained in sepsis after Day 5 with a SOFA score ≥ 2. Among them, three died within 28 days. Standard sepsis metrics were compared between these two groups. Four parameters showed significant differences: timing of sepsis to septic shock, SOFA score at admission, use of hydrocortisone and ICU stay. General characteristics of patients and their clinical outcomes related to SOFA score at Day 5 are presented in Table 1.

**Table 1:** General characteristics of patients and according to SOFA score at Day 5

Characteristics	Overall n = 39	Recovered from sepsis within 5 days n = 21 (56.4%)	Prolonged sepsis n = 18 (43.6%)	*p*-value
Age, years	67 [54–74]	64 [54–77]	63 [53–72]	0.879
Female Male	9 (23,1%) 30 (76.9%)	7 (31.8%) 14 (77.8%)	2 (11.7%) 16 (76.2%)	0.908
BMI	27.8 [22.9–31.3]	27.5 [24–31.8]	27.8 [22.5–31.2]	0.508
Immunosuppression	10 (25.6%)	5 (23.8%)	5 (27.7%)	0.853
Origin of infection				
Digestive	21 (53.8%)	11 (61.1%)	10 (47.6%)	0.727
Pulmonary	7 (17.9%)	2 (11.1%)	5 (23.8%)	
Urinary	5 (12.8%)	2 (11.1%)	3 (14.3%)	
Other	6 (15.4%)	3 (16.7%)	3 (14.3%)	
Onset of infection/ICU admission (hours)	27 [21–48]	23 [14–28]	40 [24–48]	0.026*
SOFA	9 [2–16]	6 [4–10]	10 [7–12]	0.002*
APACHE II SAPS II	19 [15 to 23] 43 [34–52]	19.5 [14–22] 40.5 [34–47]	18 [15–24] 50 [36–54]	0.405 0.215
Surgery during stay	16 (41%)	8 (36.4%)	8 (47.1%)	0.692
Use of hydrocortisone	19 (48 .7%)	6 (27.3%)	13 (76.5%)	0.017*
Positive blood cultures	21 (53.8%)	12 (54.5%)	9 (52.9%)	0.6597
Positive blood RNA 16S	12/34 (35.3%)	7/20 (35%)	5/14 (35.7%)	0.2433
ICU length of stay (days)	10 [5 to 15]	8 [5–12]	22 [11–22]	0.022*

Data are presented with percentage (%) or median [interquartile]

### Dosages and kinetics of PAMP

Of all 399 values analyzed, PAMP levels ranged from 0.5 to 37 ng/mL, with a mean of 9, a median of 5, and an interquartile range from 3 to 11.

**Fig. 1:**
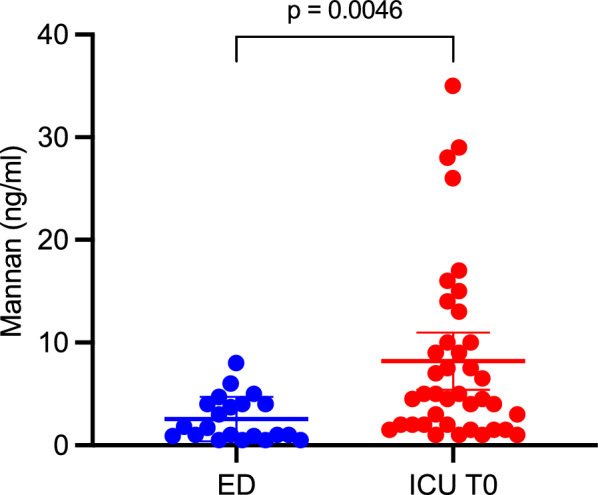
Comparison of level of PAMPs at the first dosage in ICU patients to a group of control patients (admitted to the emergency department for noninfectious conditions: trauma (n = 10), cardiologic issues (n = 5), neurologic conditions (n = 3), and obstetrical cases (n = 3). The median WBC was 8.85 G/L and median CRP 5 mg/L), 5 ng/ml versus 1.7 ng/ml

Initial median PAMP levels were significantly higher in septic shock patients than in ED controls with noninfectious conditions, 5 ng/mL versus 1.7 ng/mL (p = 0.0046) (Fig. 1).

**Fig. 2:**
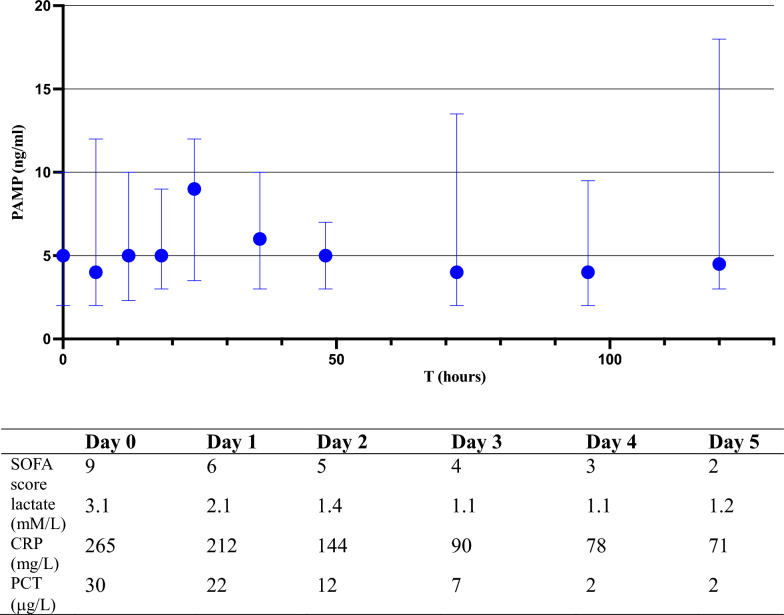
Kinetics of PAMP with median values and interquartile range of all dosages at the different sampling times for 39 patients in septic shock. Daily median SOFA score and biomarkers are listed underneath

Median peak concentration reached 9.0 ng/mL at 24 h and gradually decreased to 4.5 ng/mL by Day 5 (Fig. 2).

Importantly, we observed distinct PAMP kinetic profiles by persistence of sepsis after Day 5 defined with a SOFA score ≥ 2. At enrollment, the SOFA score had a median value of 9 (2—16). Daily SOFA scores declined faster in the short-term sepsis group and remained significantly higher in the prolonged sepsis group from Day 1 (10 versus 3) to Day 5 (7 versus 1).

**Fig. 3:**
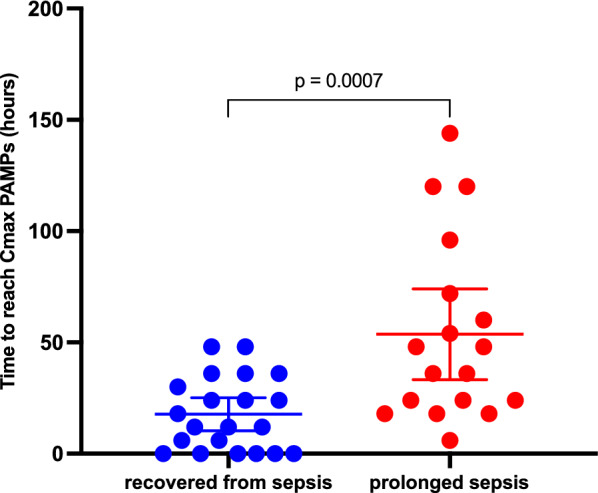
Time to reach C_max_ PAMP after admission in ICU. Recovered from sepsis at Day 5 (n = 21) 18H (10 to 24). Prolonged sepsis (n = 18) 48H (33 to 72)

Primary analysis did not reveal significant differences between patient groups. We studied in a secondary analysis the timing of concentration variations. In patients with prolonged sepsis, Tmax PAMP was significantly delayed, 48H versus 18H (p = 0.0007) (Fig. 3).

**Fig. 4:**
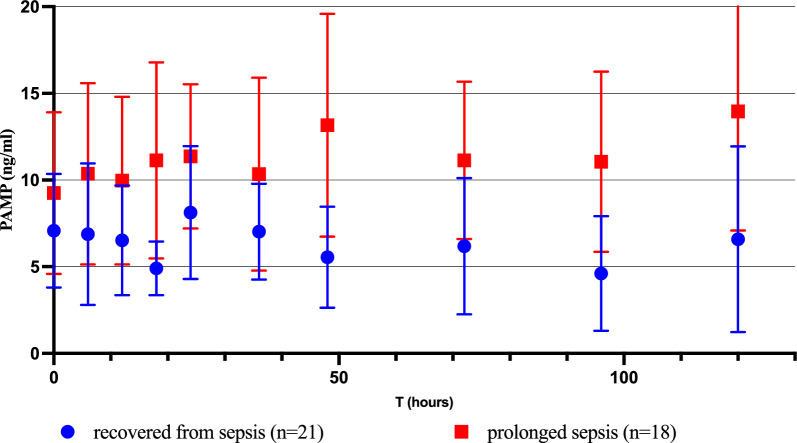
Comparison of kinetics of PAMP between patients recovered from sepsis at Day 5 and those not. Median values and interquartile range

The prolonged sepsis group also had higher AUC values and remained above the median PAMP concentration for a longer duration (71.5% vs. 21.3%; p = 0.05). These divergences became more pronounced after Hour 36 with the two groups demonstrating a different Cmax (Table 2) (Fig. 4).

**Table 2:** Serially measured values of PAMPs characteristics of their kinetics

	Serially measured variables	Recovered from sepsis at day 5 n = 21 Median (p25;p75)	Prolonged sepsis n = 18 Median (p25;p75)	p value
All dosages of PAMPs from 0 to Day 5	C_min_ PAMPs (ng/mL)	2 (1; 3)	2 (1.5; 3.9)	0.193
	C_max_ PAMPs	11.5 (7.5; 19)	12 (10; 27.8)	0.170
	T_max_ PAMPs (hours)	18 (10; 24)	48 (33; 72)	0.001*
	Time-weighted average PAMPs	4.60 (3.7; 7.8)	6.5 (4.6; 15.7)	0.114
	% Time above global median value (5 ng/mL)	21.3 (0; 74.4)	71.5 (27.2; 96.5)	0.049*
	AUC- Time Interval PAMPs	108 (72; 120)	120 (120; 120)	0.021*
Dosages from 0 to 36H	First PAMPs ≤ 36H	4 (1; 13.5)	4.5 (1; 7.9)	0.890
	Last PAMPs ≤ 36H	4 (2; 6.8)	4 (2; 10.6)	0.489
	C_min_ PAMPs ≤ 36H	2 (1; 5)	4 (1.5; 6.3)	0.175
	C_max_ PAMPs ≤ 36H	9 (3.9; 16.8)	5 (3.6; 10.9)	0.427
	T_max_ PAMPs ≤ 36H	6 (0; 13.5)	6 (0; 18)	0,999
	Time-weighted average PAMPs ≤ 36H	4.9 (2.6; 9.6)	4.6 (2.3; 9)	0.972
	% Time above global median value (5 ng/mL) ≤ 36H	29.2 (0; 100)	0 (0; 100)	0.659
Dosages from 36H to Day 5	First PAMPs > 36H	6.8 (3; 9)	4 (2.8; 10.3)	0.999
	Last PAMPs > 36H	4 (2; 7)	8 (3.4; 25.8)	0.095
	C_min_ PAMPs > 36H	2.5 (1; 4)	3 (1.5; 4.3)	0.319
	C_max_ PAMPs > 36H	9 (5; 12)	12 (9.8; 30.3)	0.025*
	T_max_ PAMPs > 36H	0 (0; 30)	24 (10.5; 69)	0.040*
	Time-weighted average PAMPs > 36H	4.2 (3.3; 6)	6.4 (4.6; 15.9)	0.020*
	% Time above global median value (5 ng/mL) > 36H	25.6 (0; 73.6)	72.9 (29.5; 96.5)	0.041*

Data are represented for all dosing schedules, and before and after Hour 36 following admission

### Secondary outcomes

**Fig. 5:**
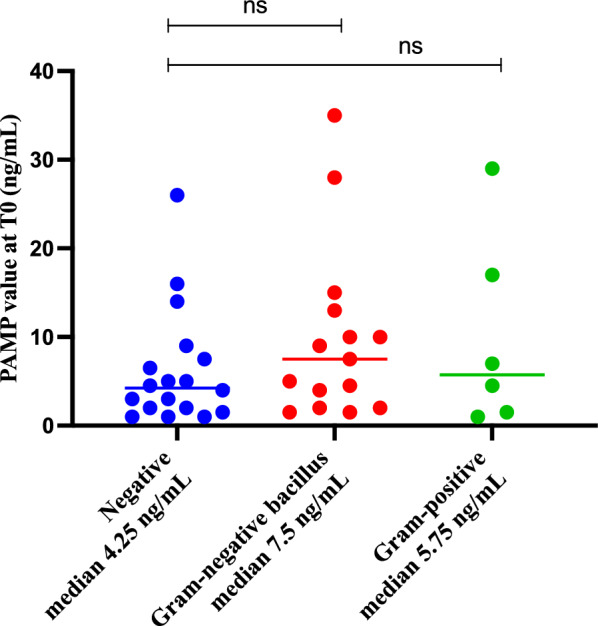
Initial value of PAMP related to results of blood culture at admission

PAMP levels were not associated with infection site or pathogen species. Patients with positive blood cultures at admission (54%) had slightly higher—but not statistically significant—initial PAMP concentrations (7.0 ng/mL versus 4.25 ng/mL; p = 0.186) (Fig. 5).

All patients were infected prior to admission and had received an initial course of antibiotics at the onset of infection. The duration of antibiotic treatment before ICU admission ranged from 2 to 120 h, with a median of 27 h. Importantly, this initial regimen was escalated to broad-spectrum antibiotics in 26 of 39 patients just hours before ICU admission.

In our analysis, we used Spearman rank correlation to assess the relationship between the duration of pre-admission antibiotics and PAMP levels during the first 24 h. While most correlations did not reach statistical significance, a notable exception was observed for H18 dosage (r = 0.39, p = 0.015) (see Additional Table 1).

Lactate levels were elevated for a longer period in the not-cured group and also showed a delayed Tmax (p = 0.03). CRP and PCT were not helpful in discriminating between our two groups of patients (Table 3).

**Table 3:** Secondary outcome criteria

Parameter	Serially measured variables	Recovered from sepsis at day 5 n = 21 Median (p25;p75)	Prolonged sepsis n = 18 Median (p25;p75)	p value
Lactate (mM/L)	First lactate (mM/L) Last lactate C_min_ lactate C_max_ lactate T_max_ lactate Time-weighted average lactate	3.8 (2.1; 4.2) 1 (0.8; 1.2) 0.9 (0.7; 1) 3.8 (2.1; 4.4) 0 (0; 0) 1.4 (1.1; 1.9)	2.5 (2.2; 3.3) 1.2 (1; 1.5) 1 (0.8; 1.3) 3 (2.3; 3.9) 0 (0; 24) 1.7 (1.5; 2.2)	0.199 0.025* 0.102 0.525 0.030* 0.195
CRP (mg/L)	First CRP (mg/L) Last CRP C_min_ CRP C_max_ CRP T_max_ CRP Time-weighted average CRP	265 (207; 346) 122 (84; 219) 122 (84; 160) 273 (217; 347) 0 (0; 0) 209 (154; 256)	291 (182; 367) 156 (74; 297) 156 (74; 297) 295 (182; 367) 0 (0; 0) 206 (117; 309)	0.612 0.849 0.568 0.949 0.059 0.812
PCT (µg/L)	First PCT (µg/L) Last PCT C_min_ PCT C_max_ PCT T_max_ PCT	32 (8; 64) 12 (3; 34) 12 (3; 34) 32 (8; 64) 0 (0; 0)	32 (7; 91) 7.2 (4.8; 62) 7.2 (4.8; 62) 32 (7; 91) 0 (0; 0)	0.848 0.787 0.787 0.898 0.596
	Time-weighted average PCT	21 (4.8; 52)	24 (7; 78)	0.849

Links analysis showed a relationship among Tmax PAMPs, Tmax lactate and Tmax CRP. Because of that linkage, respective AUCs were compared. The AUCs for a ROC for Tmax PAMPs, Tmax CRP and Tmax lactate were, respectively, 0.8 (0.63–0.91), 0.6 (0.41–0.75) and 0.62 (0.44–0.77). The most discriminative factor for an unfavorable outcome was Tmax PAMPs.

## Discussion

In this cohort study of patients in septic shock, kinetic analysis of PAMPs measured by an FcMBL ELISA independently correlated to persistence of sepsis beyond 5 days. A prolonged sepsis was associated with a PAMPs peak that exhibited a sustainable upwards trend beyond H36.

This study is one of the first investigations into the dynamics of PAMPs during human septic shock, and consequently there was limited rationale available for defining the sampling schedule [11]. Our working hypothesis was that, as a major and early trigger of the cytokine storm associated with septic shock, PAMP levels would be directly associated with sepsis severity and would therefore reach their maximum during the first 24 h. A 6-h sampling interval during the first day in the ICU was chosen as a reasonable compromise compatible with standard care. Our study demonstrated, however, that peak PAMP levels were delayed in all patients, with the largest variations occurring between Day 1 and Day 2. These findings provide important new insights and will help optimize sampling intervals for future studies.

Although baseline PAMP levels exhibited large inter-individual variations, median PAMP values in our study population were higher than those in the control group, similar to two prior clinical studies. From a cohort of infected ED patients, PAMP levels were significantly higher than in blood donors and non-infected trauma patients [9]. In burn patients, PAMP absolute values in those with sepsis differed from those without infection. Increases in PAMP levels correlated with developing sepsis [12].

Peak levels of PAMPs were delayed in all patients. Formed in infected tissues, PAMPs are released into the local microcirculation before entering the bloodstream for clearance, which could explain the delayed peak observed [13–15]. The delayed peak of PAMPs was observed in two animal studies using the same technique as ours, and similarly, PAMP elevation was observed several hours after infection. In a rat sepsis model, PAMP levels did not increase for 10 h [16]. In a pig model of septic shock, PAMP elevation started at Day 1 and peaked at Day 2 [17].

There was no direct correlation between PAMP’s level and any of the routine sepsis metrics we examined. The absence of significant differences between patients with and without positive blood cultures could be explained by the clinical records. Preadmission probabilistic antibiotherapy may have negatived the blood cultures without preventing evolution to shock. Furthermore, PAMPs are composed of bacterial debris and capture by FcMBL was enhanced by antibiotic treatment [5]. Raw PAMP's levels were more related to bacterial lysis products than to the presence of entire bacteria. In our cohort, we did not find any obvious correlation between the initiation of antibiotic treatment and the peak PAMP levels, which generally occurred approximately 24 h after ICU admission. Molecular patterns levels captured by FcMBL during septic shock exhibited large inter-patient variability, suggesting values depend on numerous parameters, and not just the time to effective antibiotic administration. This heterogeneity can be attributed to several factors, including individual host immune responses, the phase of sepsis at the time of measurement, and the adequacy of source control, particularly in surgical cases.

In blood cultures, FcMBL captures most microorganisms and PAMPs [18, 19]. But in vivo FcMBL could also capture other types of glycosylated moieties like DAMPs which are co-released during infection and bind overlapping receptors [11]. The delayed peak in severely ill patients may thus reflect not only a higher microbial burden, but a broader dysregulated immune state and persistent tissue injury, potentially driven by DAMPs released from infected organs [20].

Raw PAMP levels exhibit substantial variability during septic shock, highlighting the importance of considering their dynamics rather than absolute values. Our analysis showed that persistent elevation of PAMP levels beyond 48 h in the ICU is strongly associated with prolonged sepsis. To further explore the prognostic and therapeutic value of PAMPs, our findings indicate that a 24-h sampling interval is likely sufficient in future studies to capture the main PAMP kinetics, provided that sampling continues throughout the duration of sepsis. This would help identify patients whose levels remain elevated or continue to rise after 48 h and who might benefit from either more aggressive infection source control or targeted immunomodulatory approaches to limit the release of endogenous molecules that exacerbate the inflammatory response.

Finally, the large diversity in FcMBL-bound particles turned out to be an interesting marker, reflecting a global response. Molecular patterns measured by FcMBL expressed the complex response to infection that drives sepsis, as well as the heterogeneity between patients and within patients over time. This study demonstrated the technical feasibility and clinical reproducibility of deploying a standardized method using FcMBL ELISA.

Our study has limitations. First, no power calculation was performed. It was an observational study; the sample size was not predefined, and the relatively small number of patients is a limitation of the study. Second, although patient characteristics and PAMP kinetics were consistent in pre- and post-COVID-19 patients the 21-month interruption appeared as a temporal bias. Third, the prognostic value of PAMP was a secondary analysis which—although corrected by post hoc tests—increases the risk for false positives (type I error). Fourth, the absence of different control groups, such as healthy volunteers or critically ill non-septic patients which would provide more-defined thresholds compared to septic shock patients. And fifth, the variable sampling intervals in our study (ranging from 6 h to daily) were a limitation and may have missed subtle kinetic features.

## Conclusions

The kinetic signature of sustained or delayed PAMP elevation—particularly beyond 36 h—may represent a valuable prognostic signal and could complement existing tools in sepsis management.

Further validation in multicenter cohorts is needed, but these findings suggest that FcMBL-based measurement of molecular patterns captures the complexity of septic responses and may provide early prognostic insight in septic shock, supporting more tailored clinical decision-making.

## Data Availability

The datasets used and analyzed during the current study are available from the corresponding author on reasonable request.

## Abbreviations

AUC: Area under the curve
Cmax: Maximum concentration
CRP: C-reactive protein
DAMP: Damage-associated molecular pattern
ELISA: Enzyme-linked immunosorbent assay
ICU: Intensive care unit
IRB: International Review Board
MBL: Mannose-binding lectin
PAMP: Pathogen-associated molecular pattern
PCT: Procalcitonin
SOFA: Sequential Organ Failure Assessment
Tmax: Time to peak
